# Estimated frequency of somatic symptom disorder in general practice: cross-sectional survey with general practitioners

**DOI:** 10.1186/s12888-022-04100-0

**Published:** 2022-09-29

**Authors:** Marco Lehmann, Nadine Janis Pohontsch, Thomas Zimmermann, Martin Scherer, Bernd Löwe

**Affiliations:** 1 Department of Psychosomatic Medicine and Psychotherapy, University Medical Center Hamburg-Eppendorf, Martinistr. 52, 20246 Hamburg, Germany; 2 Department of General Practice and Primary Care, University Medical Center Hamburg-Eppendorf, Martinistr. 52, 20246 Hamburg, Germany

**Keywords:** Persistent somatic symptoms, Somatoform disorder, Primary health care, General practice, Somatic symptom disorder, Bodily distress, General practitioner

## Abstract

**Background:**

Somatic symptom disorder (SSD) is the successor diagnosis of somatoform disorder in the 5th Edition of the Diagnostic and Statistical Manual of Mental Disorders (DSM-5). Relevance and frequency of SSD and its clinical symptoms in general practice are still unknown. We estimate frequencies of patients fulfilling the diagnostic criteria of SSD in general practice.

**Methods:**

Mailed and online survey with general practitioners (GP) in Germany using a cross-sectional representative sample from registries of statutory health insurance physicians. GPs estimated percentages of their patients who show the clinical symptoms of SSD according to DSM-5; that is, one or more burdensome somatic symptoms (A criterion), excessive symptom- or illness-related concern, anxiety, or behaviour (B criterion), and persistence of the symptoms over at least 6 months (C criterion). Statistical analysis used means and confidence intervals of estimated patient proportions showing SSD symptoms. Frequency of full-blown SSD was based on the products of these proportions calculated for each GP.

**Results:**

Responses from 1728 GPs were obtained. GPs saw the clinical symptoms of SSD fulfilled (A and B criteria) in 21.5% (95% CI: 20.6 to 22.3) of their patients. They further estimated that in 24.3% (95% CI: 23.3 to 25.2) of patients, symptoms would persist, yielding a total of 7.7% (95% CI: 7.1 to 8.4) of patients to have a full-blown SSD.

**Conclusions:**

We estimate a frequency of 7.7% of patients in general practice to fulfil the diagnostic criteria of SSD. This number may figure as a reference for the yet to be uncovered prevalence of SSD and it indicates a high clinical relevance of the clinical symptoms of SSD in general practice.

**Registration:**

German Clinical Trials Register (Deutschen Register Klinischer Studien, DRKS).

DRKS-ID: DRKS00012942.

The date the study was registered: October 2nd 2017.

The date the first participant was enrolled: February 9th 2018.

**Supplementary Information:**

The online version contains supplementary material available at 10.1186/s12888-022-04100-0.

## Background

With the introduction of the 5th Edition of the Diagnostic and Statistical Manual of Mental Disorders (DSM-5) in 2013 [1], the diagnosis of somatoform disorder was replaced by somatic symptom disorder (SSD). The clinical symptoms of SSD combine both, physical and psychological symptom burden. That is, besides the A criterion of at least one life-disrupting physical symptom, SSD requires excessive symptom- or illness-related concern, anxiety, or behaviour as it’s B criterion – the so-called psychological positive criteria. Finally, SSD assumes symptom persistence of at least 6 months as its C criterion. SSD no longer requires the exclusion of a medical cause for physical complaints. The International Classification of Diseases 11th Revision (ICD-11) made a similar development from the group of somatoform disorders in ICD-10 to the diagnosis of bodily distress disorder, which is comparable with SSD [2].

Representative and unbiased prevalence studies for SSD are still rare as evidenced in a recent scoping review [3]. In a community health centre one rather small study, using a clinical interview to diagnose SSD with 202 participants, revealed 20.8% of patients diagnosed with SSD [4]. Prevalence estimation using a combination of Patient Health Questionnaire (module for somatic symptom severity, PHQ-15), Whiteley-Index, and Brief Illness Perception Questionnaire (BIPQ) as a proxy measure for SSD revealed a proportion of 45.5% of SSD in a selected sample of 325 patients with medically unexplained physical symptoms in general practice [5]. More gold standard prevalence data is still missing and there is no knowledge about what proportions of patients in clinical practice show symptoms of SSD.

With SSD also came the scholarly debate of the harms and benefits of this new diagnosis in comparison with its predecessor [6]. Some argue that SSD was an overdue invention to end dualistic thinking of mind and body in clinical practice [7, 8]. Furthermore, clinicians themselves said that they could not assess the former criterion of exclusion of physical disease with certainty anyway [9]. Overall, regarding reliability, validity, and clinical usefulness SSD seems to surpass its predecessor [3, 7, 10]. Nonetheless, it is unclear of what it means for general practice. It was designed to be viable to implement in general practice [11] and for general practitioners (GPs), who encounter many patients with persistent somatic symptoms of unclear origin. It is uncertain whether GPs evaluate the introduction of SSD as useful and in which proportion of their patient‘s they see the clinical symptoms of SSD. Furthermore, compared with its predecessor somatoform disorder, it is unclear whether GPs see continuity in the diagnoses as they apply to patients showing persistent somatic symptoms. Answering these empirical questions would help to form reasonable expectations about the relevance of SSD in general practice. It would also help to plan for the necessary resources for those in need.

Our research questions were:

1. What are the estimated proportions of patients showing the clinical symptoms of SSD and full-blown somatic symptom disorder as viewed by GPs?
2. Which variables are associated with the estimated proportions of patients showing clinical symptoms of SSD (i. e., proportion of patients with somatoform disorders, characteristics of the general practitioners and the practice setting)?

## Methods

We invited a representative sample of GPs to participate in a survey regarding the proportions of patients with somatic symptom disorder in general practice. This study was part of the project ‘*Identification of barriers and difficulties involved in the process of diagnosing somatic symptom disorders in primary care* (BeSSD-GP)’, which was funded by the German Research Foundation (Deutsche Forschungsgemeinschaft, DFG). Details of the project were published in the study protocol [12]; and the study method of this cross-sectional representative and anonymous survey is further described elsewhere [13]. We administered a questionnaire with GPs in eight federal states of Germany from February 9th to May 15th, 2018 (Registration: October 2nd, 2017, German Clinical Trials Register (DRKS00012942)).

### Participants

GPs were randomly sampled (*n* = 12,004) from all working GPs from the German federal states of Schleswig-Holstein, Hamburg, North Rhine, Saarland, Brandenburg, Saxony, Mecklenburg-Vorpommern, and Thuringia. The selection procedure was stratified random sampling with the federal states serving as strata. We sampled via publicly accessible contact information through registries of statutory health insurance physicians proportionally to the total number of general practitioners working in the respective federal states. A programmed R script randomly selected entries from the aggregated data tables consisting of the contact information. The selected GPs and their contact information were then passed on to serial letters, so that each GP in the sample could obtain a personalized invitation to participate (see data acquisition). The serial letters were produced and sent by a printing company. Eligible were physicians working in general practice. Participation was voluntary and anonymous. To comply with common research practice in Germany, we offered no incentive.

### Survey

The survey was administered in paper-and-pencil and online format. The paper-and-pencil version was sent via mail, the online format was announced via mail with a postcard. Because GPs may not have been familiar with the diagnostic innovation of SSD, we included a short description of the disorder prior to the questionnaire items. It referred to the publication of DSM-5, to the introduction of positive psychological criteria for SSD (B criteria), and to the abortion of the diagnosis by exclusion of physical illness.

GPs assessed the following items:

- To what extent they evaluate the introduction of SSD as meaningful
- The frequency of occurrence (as percentage of all patients) of fulfilled diagnostic criteria
- ◦ A: One or more somatic symptoms that are distressing or result in significant disruption of daily life.
- ◦B1: Disproportionate and persistent thoughts about the seriousness of one’s symptoms.
- ◦ B2: Persistently high level of anxiety about health or symptoms.
- ◦ B3: Excessive time and energy devoted to these symptoms or health concerns.
- ◦ C: Although any one somatic symptom may not be continuously present, the state of being symptomatic is persistent (typically more than 6 months).
- ◦ Combinations of A- and B-criteria of SSD in their practice.
- Person characteristics
- ◦ Gender
- ◦ Years of practice
- Practice characteristics
- ◦ Practice setting: own practice or collaborative practice
- ◦ Practice region: rural, small town, medium town, large town
- ◦ Number of patients per quarter
- Estimated proportion of patients with Diagnostic and Statistical Manual of Mental Disorders (4th edition, text revision, DSM-IV-TR) somatoform disorders (%) [14].

We captured the subjective meaningfulness of the SSD innovation with a six-point Likert rating scale between *1: does not apply* to *6: applies completely*. We presented the items regarding the subjective frequency of diagnostic categories as Likert rating scales with eleven response options ranging from 0% in steps of 10 to 100%. So, the frequency estimations for the diagnostic categories of SSD are solely based on the assessments of the GPs and are not based on standardized measurements with patients. Person and practice characteristics used categorical ratings. Years of practice and estimated proportion of patients with somatoform disorder asked for provision of a single number.

### Data acquisition

We invited GPs to participate by mail or online. They received a post card announcement and two consecutive mailings of the survey package. Data acquisition commenced February 9th, 2018 with the announcement 1 week prior the first mailing of the survey, with the second mailing following 2 weeks later. Data collection was closed on May 15th, 2018. There was also an option for online participation. The invitation to participate consisted of a data safety statement, assurance of anonymous data analysis, and statement that participation was voluntary. Due to the completely anonymous data collection, no written informed consent was necessary in accordance with German law. GPs were notified that informed consent would be presumed if they returned the completed response form.

### Statistical analysis

All analyses were conducted in R (Version 3.6.2). For this data the analysis of responders and non-responders and the comparison of the sample characteristics with official statistics is provided elsewhere [13]. We analysed the item of how the GPs appraised the meaning of the diagnostic innovation and the six items regarding the clinical symptoms of SSD in general practice and present means and 95% confidence intervals (95% CI) for these items. An estimate of the frequency of SSD in general practice was calculated using a derived variable, which was the product of the proportions, calculated for each GP, of patients showing the clinical symptoms of SSD (A and B criteria) and the proportion of patients showing symptom persistence (C criterion). Furthermore, we used ordinal regression to model the estimated proportions of patients showing the clinical symptoms of SSD. The regressions used person and practice characteristics and the estimated proportion of patients with DSM-IV-TR somatoform disorders as independent variables. Regressions were calculated with the *ordinal* R package [15]. All tests for significance were two-tailed. Multicollinearity between predictors was checked using variance inflation factor from the *car* R package [16].

## Results

We obtained responses from 1829 GPs (15.2%). Reasons for non-participation were the retirement or death of the GP, comments that the survey method would be inappropriate, and non-specified reasons. After removal of cases with double participation and cases with empty person characteristics, we analysed the data of 1728 general practitioners. The participant and practice characteristics shown in Table 1.

**Table 1:** Participant characteristics

	Category	Sample
n		1728
Gender (%)	male	760 (44.8)
	female	938 (55.2)
Years of practice (%)	0–10	455 (27.4)
	11–20	540 (32.5)
	21–30	454 (27.3)
	> 30	211 (12.7)
Practice setting (%)	Own practice	837 (50.1)
	Collaborative practice	834 (49.9)
Practice region (%)	Rural (max. 4.999 Inh.)	321 (19.6)
	Small town (5.000 to 19.999 Inh.)	369 (22.5)
	Medium town (20.000 to 99.999 Inh.)	351 (21.4)
	Large town (above 100.000 Inh.)	600 (36.6)
Number of patients per quarter (%)	< 500	53 (3.4)
	500–1000	654 (42.4)
	1001–1500	647 (41.9)
	> 1500	190 (12.3)
Estimated proportion of patients with DSM-IV-TR^a^ somatoform disorders (%)	0%	210 (15.2)
	10%	524 (37.8)
	20%	324 (23.4)
	30%	207 (14.9)
	> 30%	120 (8.7)

^a^*DSM-IV-TR* 4th edition, text revision, Diagnostic and Statistical Manual of Mental Disorders

**Fig. 1:**
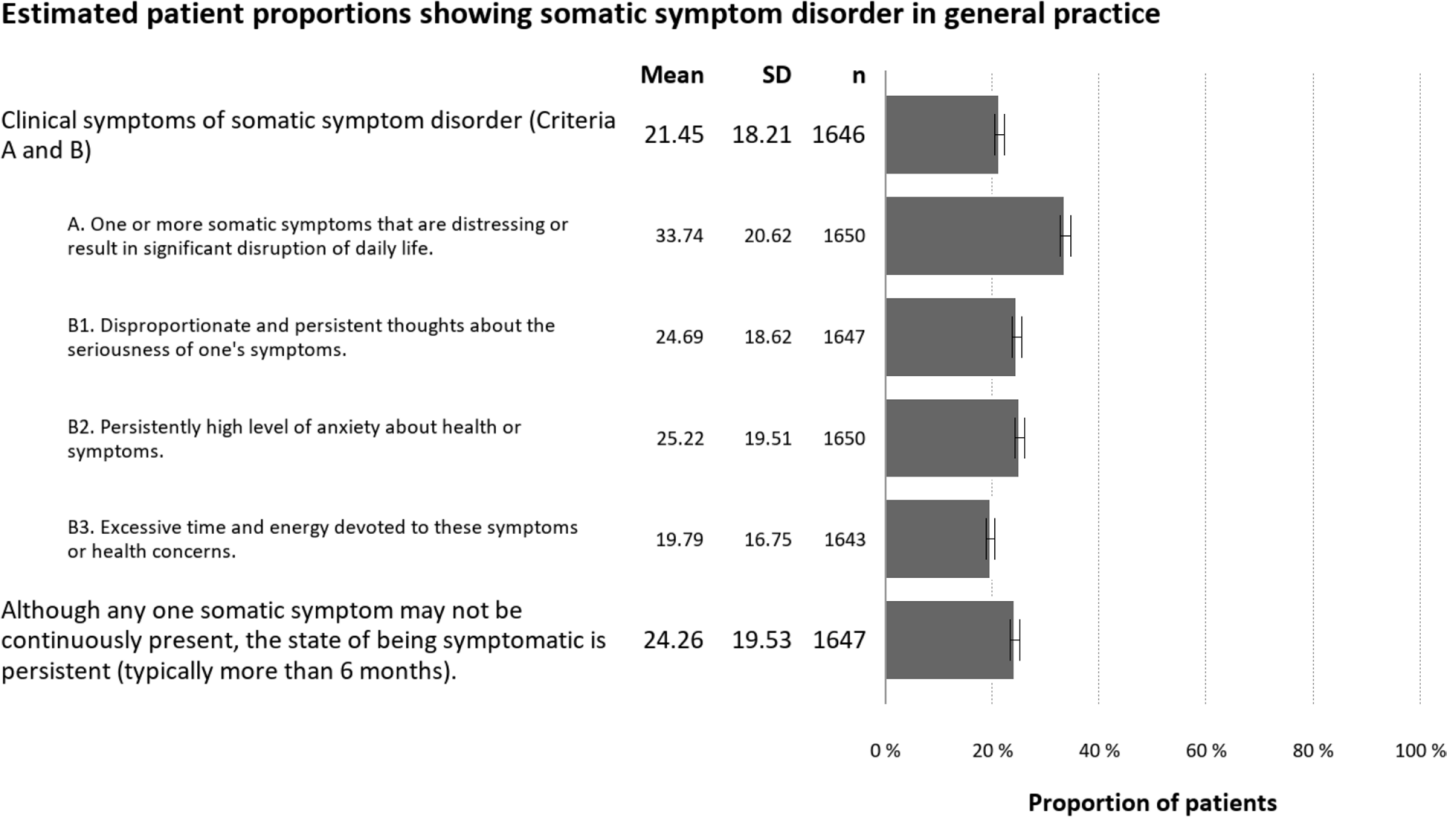
Estimated proportions of patients fulfilling clinical symptoms of somatic symptom disorder

The respondents in large part found the replacement of somatoform disorder by somatic symptom disorder meaningful (M = 4.29, 95% CI: 4.24 to 4.35). That is, the mean value reveals that the sample of general practitioners tended towards the assertion that the changes in diagnostic criteria as implemented in somatic symptom disorder are meaningful. Figure 1 shows the mean proportions of the estimated proportions for the clinical symptoms of SSD. The GPs estimated that 21.5% (95% CI: 20.6 to 22.3) of their patients would show the clinical symptoms of SSD (A and B criteria). In 24.3% (95% CI: 23.3 to 25.2) of their patients, the GPs indicated symptom persistence (C criterion). Regarding the individual diagnostic criteria, the GPs indicated that 33.7% (95% CI: 32.7 to 34.7) of patients would show one or more distressing and significantly impairing somatic symptoms (A criterion). Furthermore, they estimated excessive levels of symptom related thoughts, anxiety, and behaviour in 24.7% (95% CI: 23.8 to 25.6), 25.2% (95% CI: 24.3 to 26.2), and 19.7% (95% CI: 19.0 to 20.6) of their patients, respectively (B criteria). The new variable, consisting of the case wise product of the estimated proportion of patients with clinical symptoms of SSD (A and B criteria), and the estimated proportion of patients with symptoms persisting for more than 6 months (C criterion), yielded a mean frequency estimate of 7.7% (95% CI: 7.1 to 8.4) of patients in general practice with a diagnosis of full-blown SSD.

For the ordinal regression analyses, to obtain enough counts in all outcome categories, all estimated proportions of diagnostic criteria higher than 40% were aggregated into one category. Variance inflation factors for all predictors in all analyses indicated that multicollinearity was not problematic (VIF < 1.1). The regression analyses indicated that the proportion of patients showing a somatoform disorder was the only significant predictor for the proportions of patients showing the clinical symptoms of SSD (Table 2). All other person and practice characteristics were not significantly associated with clinical symptoms of SSD. Significant Spearman rank correlations (*r*_*S*_ = 0.36 to 0.41, *p* < 0.001) confirmed that higher estimated proportions of patients showing SSD symptoms were associated with higher estimated proportions of patients showing a somatoform disorder. We further recalculated the analyses as standard linear regressions with quantitative outcomes and the ordinal regressions with data imputations to include higher sample sizes. Both auxiliary analyses yielded the same pattern of results as the initial ordinal regression.

**Table 2:** Regression coefficients and standard errors of ordinal regression of SSD diagnostic criteria regarding participant characteristics, practice characteristics, and the estimated proportion of patients with somatoform disorder

	Clinical symptoms of somatic symptom disorder					
	Criteria A and B both apply	One or more somatic symptoms (A)	Persistent thoughts about symptoms (B1)	Persistent anxiety about symptoms (B2)	Excessive time and energy spent for symptoms (B3)	Symptom persistence (C)
	B (SE)	B (SE)	B (SE)	B (SE)	B (SE)	B (SE)
Gender female	−0.04 (0.11)	0.05 (0.11)	−0.08 (0.11)	− 0.12 (0.11)	− 0.03 (0.11)	0.07 (0.11)
11–20 years of practice	− 0.15 (0.15)	− 0.17 (0.14)	−0.38^**^ (0.14)	− 0.14 (0.14)	−0.26 (0.15)	− 0.24 (0.14)
21–30 years of practice	−0.11 (0.16)	− 0.18 (0.15)	−0.22 (0.15)	0.08 (0.15)	−0.20 (0.16)	− 0.29 (0.15)
> 30 years of practice	− 0.15 (0.19)	−0.45^*^ (0.18)	− 0.35 (0.18)	0.06 (0.19)	− 0.21 (0.19)	−0.36 (0.19)
Collaborative practice	−0.09 (0.11)	0.10 (0.11)	0.07 (0.11)	−0.03 (0.11)	−0.15 (0.12)	0.05 (0.11)
Small town (5.000 to 19.999 Inh.)	−0.35^*^ (0.17)	−0.11 (0.17)	− 0.07 (0.17)	−0.31 (0.17)	− 0.15 (0.17)	−0.16 (0.17)
Medium town (20.000 to 99.999 Inh.)	0.01 (0.17)	0.02 (0.17)	−0.15 (0.17)	−0.16 (0.17)	− 0.04 (0.18)	0.08 (0.17)
Large town (above 100.000 Inh.)	−0.10 (0.16)	0.07 (0.16)	−0.04 (0.16)	0.05 (0.16)	0.07 (0.16)	0.08 (0.16)
More than 1000 patients per quarter	0.03 (0.11)	−0.14 (0.11)	0.14 (0.11)	0.22^*^ (0.11)	−0.05 (0.12)	−0.09 (0.11)
Proportion of patients showing a DSM-IV-TR somatoform disorder	2.33^***^ (0.17)	1.89^***^ (0.15)	1.91^***^ (0.16)	2.01^***^ (0.16)	1.87^***^ (0.16)	1.96^***^ (0.16)
Observations	1131	1129	1130	1130	1125	1129
Log Likelihood	− 1579.15	− 1746.65	− 1671.43	− 1673.78	− 1564.65	− 1701.27

Note:^*^p < 0.05; ^**^p < 0.01; ^***^p < 0.001

Predictor reference categories: Gender male, 0–10 years of practice, own practice, rural practice region (max. 4.999 Inh.), 0–1000 patients per quarter

## Discussion

GPs in our study had estimated that about one fifth of their patients show clinical symptoms of SSD related to the main A and B criterion; namely life-disrupting physical symptoms and excessive symptom- or illness-related thoughts, concern, and behaviour. Moreover, symptom persistence of more than 6 months was reported for 24.3% of patients – C criterion. The assessments for all diagnostic criteria combined, indicated that a frequency of 7.7% patients in general practice were likely to possess full-blown SSD. Furthermore, GPs estimated that 34% of their patients would show one or more distressing symptoms for the diagnostic A criterion of SSD, and between 20 and 25% of their patients would fulfil any one of the B criteria. The estimated proportion of patients showing a somatoform disorder (according to DSM-IV-TR) best predicted the proportions of clinical symptoms of SSD.

Although, our data does not consist of the prevalence rates estimated based on standard clinical interviews, our frequency estimation of 7.7% can be compared with estimations from other research. A small sample study in general practice yielded a prevalence of 20.8% for SSD [4]. In the general population, the prevalence of SSD was estimated to be 4.5% [17]. About 20% of the general population were at least sometimes concerned about bodily symptoms, had higher levels of anxiety related to symptoms, or showed higher levels of symptom-related behaviour [18]. In conclusion, our estimation of SSD frequency is lower than earlier data from general practice, but corresponds with estimations for the general population of about one out of twenty persons to show SSD.

### Strengths and limitations

A strength of our study is the large representative sample. We were able to randomly select from comprehensive registries of GPs in eight federal states of Germany. Moreover, we collected completely anonymous responses and informed the invited GPs about this to reduce the likelihood of socially desirable answers. So, all participants could respond based on their personal views. A further strength of our study is that it supplies timely results about patient proportions in general practice to inform health care planning when prevalence data for SSD is still elusive. Prevalence estimations require conduct of a standardized clinical interview with a large representative patient sample. Such research has not been published up to now. Our study is limited by the still unsatisfactory dissemination of the SSD concept in clinical practice in Germany. In the questionnaire, we introduced the concept of SSD in a short paragraph, so that all participants had at least minimal information about the diagnosis. Furthermore, we used literal translations of the SSD criteria, which may also be intelligible without extra information. However, it was possible that in our questionnaire some participants learned about SSD for the first time. Our study is further limited by the use of subjective estimations regarding patient proportions by the GPs. We did not ask them to search through their actual clinical records for SSD cases, because this would have limited our sample to those patients who were formally diagnosed with SSD.

## Conclusions

We estimate a frequency of 7.7% of patients in general practice to fulfil the diagnostic criteria of SSD. Even if this result is based on subjective frequency estimations by GPs and is not a prevalence, it may figure as a reference for the yet to be determined prevalence of SSD. Furthermore, we found the strongest association between the estimated patient proportions of those showing the clinical symptoms of SSD and those with somatoform disorder. This suggests that, presently, GPs may treat the diagnostic criteria of SSD as a continuation of somatoform disorder as it applies to patients showing persistent somatic symptoms and symptom-related concern. So, the results may be due to the actual overlap of both diagnoses in patients in general practice.

With passing time the diagnosis of SSD as a mental disorder may become more and more familiar not only for psychological or psychiatric specialists but also for GPs. Its implementation in similar form in ICD-11 may further acquaint clinicians who do not use DSM-5 to diagnose. Alertness for patients suffering from SSD in daily routine should rise. It is, therefore, desirable to follow-up on the present research with a longitudinal perspective to study whether knowledge and application of SSD grows. This might be particularly noteworthy in the comparison of health-care systems. If we find that knowledge and application of SSD in general practice are far behind its actual implementation in the health-care systems, training for GPs should be devised and evaluated.

It is clear that valid data about the prevalence of SSD in general practice requires representative sampling and a diagnosis based on a clinical interview as gold standard. Until we have these numbers, we have to be content with hopefully converging evidence from different sources, for example, from proxy diagnoses via psychometric tests or from non-random and convenience samples. In this actual state of the evidence, our estimations by GPs about how often the clinical symptoms of SSD occur in general practice are an important component. No matter of what eventually turns out to be the best estimate of the prevalence of SSD, the clinical assessments of GPs are decisive to initiate further treatment of patients. Since many participating general practitioners may not have been familiar with the diagnostic concept of somatic symptom disorder prior to the study, the estimate of 7.7% of full-blown SSD in general practice is probably a conservative estimate. Nevertheless, this frequency also shows that SSD must be regarded as a frequent and relevant clinical picture in general practice, which should not be underestimated or ignored diagnostically. Thus, it is not enough to introduce the diagnosis, but to disseminate it so that clinicians at gatekeeper positions might take the correct steps for further diagnosis and treatment of the afflicted patients.

## Supplementary Information

**Additional file 1.**

## Data Availability

The datasets generated during and analyzed during the current study are not publicly available because this step was not covered by approval of the ethics committee and funding agency, but are available from the corresponding author on reasonable request. The datasets used and/or analysed during the current study are available from the corresponding author on reasonable request.

## Abbreviations

SSD: Somatic symptom disorder
DSM-5: Diagnostic and Statistical Manual of Mental Disorders, 5th edition
GP: General practitioner
ICD-11: International Statistical Classification of Diseases, 11th edition
ICD-10: International Statistical Classification of Diseases, 10th edition
DSM-IV-TR: Diagnostic and Statistical Manual of Mental Disorders Text Revision
PHQ-15: Patient Health Questionnaire
BIPQ: Brief Illness Perception Questionnaire
BeSSD-GP: Identification of barriers and difficulties involved in the process of diagnosing somatic symptom disorders in primary care
VIF: Variance inflation factor
CI: Confidence interval
