# Supplementary material for: Estimated frequency of somatic symptom disorder in general practice: cross-sectional survey with general practitioners
Source: BMC Psychiatry. 2022 Sep 29;22:632. doi: 10.1186/s12888-022-04100-0 (PMC9524044; doi:10.1186/s12888-022-04100-0)
Supplement: Supplementary file 1 — Additional file 1. [file 12888_2022_4100_MOESM1_ESM.docx]

# Estimated frequency of somatic symptom disorder in general practice: cross-sectional survey with general practitioners

Marco Lehmann^a^, Nadine Janis Pohontsch^b^, Thomas Zimmermann^b^, Martin Scherer^b^, Bernd Löwe^a^

^a^Department of Psychosomatic Medicine and Psychotherapy, University Medical Center Hamburg-Eppendorf, Martinistr. 52, 20246 Hamburg, Germany

^b^Department of General Practice and Primary Care, University Medical Center Hamburg-Eppendorf, Martinistr. 52, 20246 Hamburg, Germany

# Supplementary material. Introduction and items relating to somatic symptom disorder

| **Introduction (German)** | **Translation of the introduction** |
| --- | --- |
| Ihre Einschätzung zum neuen Konzept der somatischen Belastungsstörung:  In der neuen Version des führenden psychiatrischen Klassifikationssystems in den USA, DSM-5 (Diagnostic and Statistical Manual of Mental Disorders der American Psychiatric Association) wurde eine Änderung des Konzeptes für die Diagnostik somatoformer Störungen (DSM-IV) hin zur somatischen Belastungsstörung (DSM-5) vorgenommen. Musste bis dato der Ausschluss einer somatischen Ursache erfolgen, um die Diagnose einer somatoformen Störung zu stellen, so kann nun die somatische Belastungsstörung über sogenannte Positivkriterien diagnostiziert werden. Dabei handelt es sich um übermäßige Sorgen und Ängste hinsichtlich der Symptome sowie einer exzessiven zeitlichen Beschäftigung mit ihnen. Wir möchten Ihnen im Folgenden Fragen zu diesem neuen Konzept der somatischen Belastungsstörung des DSM-5 stellen. | Your assessment of the new concept of somatic symptom disorder:  In the new version of the leading psychiatric classification system in the USA, DSM-5 (Diagnostic and Statistical Manual of Mental Disorders of the American Psychiatric Association), a change was made in the concept for the diagnosis of somatoform disorders (DSM-IV) to somatic symptom disorder (DSM-5). Until now, a somatic cause had to be excluded in order to make a diagnosis of somatoform disorder, but now somatic symptom disorder can be diagnosed using so-called positive criteria. These are excessive worries and fears about the symptoms as well as an excessive temporal preoccupation with them. In the following, we would like to ask you questions about this new concept of somatic symptom disorder of the DSM-5. |
| **Original item (German)** | **Item translation** |
| Ich halte die oben beschriebene Veränderung der Diagnosekriterien von der somatoformen Störung, hin zur somatischen Belastungsstörung für sinnvoll. | I think that the change in the diagnostic criteria from somatoform disorder to somatic stress disorder described above makes sense. |
| Welcher Anteil Ihrer Patient/innen hat eines oder mehrere somatische Symptome, die belastend sind und zu erheblichen Einschränkungen in der alltäglichen Lebensführung führen? | What proportion of your patients have one or more somatic symptoms that are distressing and lead to significant limitations in their daily living? |
| Welcher Anteil Ihrer Patient/innen hat unangemessene und andauernde Gedanken bezüglich der Ernsthaftigkeit der vorliegenden Symptome? | What proportion of your patients have inappropriate and persistent thoughts about the seriousness of their symptoms? |
| Welcher Anteil Ihrer Patient/innen hat anhaltend stark ausgeprägte Ängste in Bezug auf die Gesundheit oder die Symptome? | What proportion of your patients have persistent and severe anxiety about their health or symptoms? |
| Welcher Anteil Ihrer Patient/innen betreibt einen exzessiven Aufwand an Zeit und Energie für die Symptome oder Gesundheitssorgen? | What proportion of your patients spend excessive time and energy on symptoms or health concerns? |
| Bei welchem Anteil Ihrer Patient/innen ist die Symptombelastung persistierend (> 6 Monate, einzelne Symptome müssen nicht durchgängig vorhanden sein)? | In what proportion of your patients is the symptom burden persistent (> 6 months, individual symptoms need not be continuous)? |
| Bei welchem Anteil Ihrer Patient/innen treten anhaltende, belastende und einschränkende somatische Symptome gemeinsam mit andauernden Gedanken, Ängsten und Zeitaufwand bezüglich dieser Symptome auf? | What proportion of your patients experience persistent, distressing and limiting somatic symptoms together with persistent thoughts, anxieties and time spent on these symptoms? |
